# DesiRNA: structure-based design of RNA sequences with a replica exchange Monte Carlo approach

**DOI:** 10.1093/nar/gkae1306

**Published:** 2025-01-20

**Authors:** Tomasz K Wirecki, Grzegorz Lach, Nagendar Goud Badepally, S Naeim Moafinejad, Farhang Jaryani, Gaja Klaudel, Kalina Nec, Eugene F Baulin, Janusz M Bujnicki

**Affiliations:** Laboratory of Bioinformatics and Protein Engineering, International Institute of Molecular and Cell Biology in Warsaw, ul. Ks. Trojdena 4, 02-109 Warsaw, Poland; Laboratory of Bioinformatics and Protein Engineering, International Institute of Molecular and Cell Biology in Warsaw, ul. Ks. Trojdena 4, 02-109 Warsaw, Poland; Institute of Theoretical Physics, Faculty of Physics (FUW), University of Warsaw, ul. Hoża 69, 00-681 Warsaw, Poland; Laboratory of Bioinformatics and Protein Engineering, International Institute of Molecular and Cell Biology in Warsaw, ul. Ks. Trojdena 4, 02-109 Warsaw, Poland; Laboratory of Bioinformatics and Protein Engineering, International Institute of Molecular and Cell Biology in Warsaw, ul. Ks. Trojdena 4, 02-109 Warsaw, Poland; Laboratory of Bioinformatics and Protein Engineering, International Institute of Molecular and Cell Biology in Warsaw, ul. Ks. Trojdena 4, 02-109 Warsaw, Poland; Laboratory of Bioinformatics and Protein Engineering, International Institute of Molecular and Cell Biology in Warsaw, ul. Ks. Trojdena 4, 02-109 Warsaw, Poland; Institute of Theoretical Physics, Faculty of Physics (FUW), University of Warsaw, ul. Hoża 69, 00-681 Warsaw, Poland; Laboratory of Bioinformatics and Protein Engineering, International Institute of Molecular and Cell Biology in Warsaw, ul. Ks. Trojdena 4, 02-109 Warsaw, Poland; Laboratory of Bioinformatics and Protein Engineering, International Institute of Molecular and Cell Biology in Warsaw, ul. Ks. Trojdena 4, 02-109 Warsaw, Poland; Laboratory of Bioinformatics and Protein Engineering, International Institute of Molecular and Cell Biology in Warsaw, ul. Ks. Trojdena 4, 02-109 Warsaw, Poland

## Abstract

Designing RNA sequences that form a specific structure remains a challenge. Current computational methods often struggle with the complexity of RNA structures, especially when considering pseudoknots or restrictions related to RNA function. We developed DesiRNA, a computational tool for the design of RNA sequences based on the Replica Exchange Monte Carlo approach. It finds sequences that minimize a multiobjective scoring function, fulfill user-defined constraints and minimize the violation of restraints. DesiRNA handles pseudoknots, designs RNA–RNA complexes and sequences with alternative structures, prevents oligomerization of monomers, prevents folding into undesired structures and allows users to specify nucleotide composition preferences. In benchmarking tests, DesiRNA with a default simple scoring function solved all 100 puzzles in the Eterna100 benchmark within 24 h, outperforming all existing RNA design programs. With its ability to address complex RNA design challenges, DesiRNA holds promise for a range of applications in RNA research and therapeutic development.

## Graphical Abstract

**Graphical Abstract:**
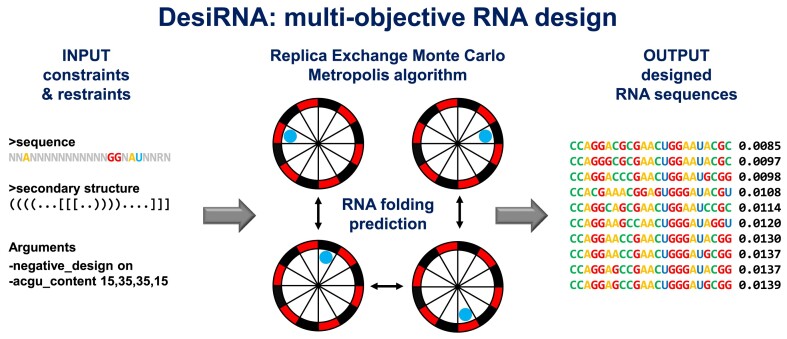

## Introduction

Ribonucleic acid (RNA) molecules represent integral components of biological systems, fulfilling crucial functions across a multitude of biological processes in living organisms. From transmitting genetic information from DNA to proteins, to sensing and communicating responses to cellular and extracellular signals, to catalyzing chemical reactions, the versatile functionality of RNA is indisputable (1). RNA molecules can also be used as potent biotechnology instruments, finding wide-ranging applications that span the development of RNA-based vaccines and therapeutics, the construction of biosensors, gene regulators and components of the gene-editing machinery. The molecular and cellular activities of an RNA molecule often necessitate it to fold into a specific structure to perform its function. The RNA sequence determines the formation of canonical base pairs that form the secondary structure, and subsequently contribute to the formation of a three-dimensional structure of the molecule. Some RNA molecules form unique structures, while others form alternative structures or undergo transitions between the structured and unstructured states (2).

There are many computational tools to predict secondary structure for individual RNA sequences or for RNA sequence alignments (3,4). Predicting RNA structure for a single sequence often assumes that the native RNA structure corresponds to the one in the global free energy minimum. The prediction is often achieved by dynamic programming, which involves constructing a matrix where each entry represents the energy calculated for individual base pairs and loops, based on experimentally derived thermodynamic parameters, filling the matrix by considering possible base pairs, and tracing back to determine the global minimum free energy (MFE) structure. These methods are deterministic and ensure the identification of the structure with the lowest free energy, as seen in RNAfold from the ViennaRNA package (5). In addition to dynamic programming, other types of methods, e.g., machine learning, have been applied for RNA secondary structure prediction. An issue with examining all conformations of an RNA molecule is that an RNA of length *n* can have approximately n^-3/2^*1.8^n^ possible secondary structures (6). The secondary structure includes not only nested pairs but also non-nested pairs forming pseudoknots. Many prediction methods exclude non-nested pairs for computational speed, but some, like IPknot (7), enable the prediction of pseudoknotted structures.

Understanding the molecular basis of RNA functions, especially those extending beyond protein coding, requires an in-depth understanding of RNA structure [review (8)]. Consequently, one of the grand challenges that synthetic biology faces is the computational design of RNA molecules with desired spatial structures and functional attributes. The significance of this challenge is reflected in the numerous applications that RNA design could enhance, ranging from the development of improved RNA vaccines, modifying HIV-1 replication mechanisms (9) and reprogramming cellular behavior (10), to developing novel logic circuits (11) and constructing intricate RNA nano-objects (12). Moreover, artificially designed RNA sequences can be exploited as tools for the detection of novel naturally occurring RNAs (13) and other molecules.

The computational design of RNA calls for solutions to the so-called RNA inverse folding problem, defined as designing a sequence that folds into a predetermined desired/target secondary structure under specific conditions. The complexity of this problem is exacerbated by the exponential growth in the number of possible sequences matching a given structure (6*^p/2^*4*^u^* for an RNA having respectively *u* unpaired and *p* paired residues) (14,15), which renders a brute force search strategy impractical. Instead, efficient search strategies usually involve mutating a seed sequence iteratively and predicting the secondary structure of each variant until a sequence that matches the desired structure is obtained. Computational RNA design algorithms are guided by fitness functions, which determine the quality of a potential solution with reference to a desired target structure (16). The seminal work of Dirks *et al.* (17) defined two paradigms for designing RNA structures, namely, the positive and negative design paradigms. While positive design seeks a sequence with high affinity for the desired structure, negative design aims for a sequence exhibiting high specificity for the desired structure. To be successful, RNA design algorithms should preferably satisfy the objectives of both paradigms.

Various algorithms have been implemented to facilitate the search for RNA sequences that fulfill the design criteria (15). Many methods allow the user to specify additional constraints or restraints, such as GC content, and the presence or absence of certain sequence patterns. Some of the recently developed methods are geared towards more specific problems, such as designing RNA sequences that fold into multiple desired structures [e.g., RNAdesign (18)], designing RNA sequences in the context of a specific 3D structure (19) or designing interacting RNA sequences (20). One of the earliest methods developed for RNA design was RNAinverse (5), which employed a simple Monte Carlo (MC) scheme (see ‘Materials and methods’ section for details). This method involved iteratively mutating an initial sequence and evaluating its folding using a cost function that minimized the ‘structure distance’ between the test sequence and the target structure.

An experiment known as Eterna has been established to challenge computer algorithms and human participants with various RNA design puzzles (21). It has provided a comparative benchmark Eterna100 and provided insight into structural features that govern the ‘designability’ of individual RNA structures as well as of structural switches. The Eterna100 dataset serves as a highly challenging benchmark for evaluating RNA design methods due to its unique characteristics and difficulties. It encompasses a diverse array of RNA secondary structures, ranging from simple hairpins to highly intricate motifs. The dataset includes sequences of varying lengths (12–400 nucleotides) and features motifs known to challenge computational methods, such as short stems, large internal loops, multiloops and zigzag patterns. The longest design targets often exhibit symmetry and repetitive elements, increasing the risk of mispairing and complicating successful sequence design. The Eterna100 benchmark was curated through the Eterna community, using systematic tests with human experts and multiple algorithms, ensuring that it spans a wide range of design difficulties and highlights the limitations of existing methods. While specific sequence characteristics like GC content are not explicitly defined, the focus on structurally challenging elements makes Eterna100 a standard for testing any method’s capability to generate RNA sequences that fold into target secondary structures.

The one minute, one hour and 24 h limits for solving puzzles of the Eterna benchmark have been introduced by researchers employing the Eterna100 benchmark to evaluate the performance of their RNA design algorithms, and they have been used consistently in the literature. Thus far, no fully automated method was able to solve all puzzles in the Eterna100 benchmark within 24 h. To this day, the best performers were: MoiRNAiFold (22), ES + eM2dRNAs (23), and NEMO (NEsted MOnte Carlo RNA puzzle solver) (24), which solved 91, 94, and 95 puzzles in 24 h, respectively, leaving two puzzles (#97 and #100) so far unsolved by any of the automated methods. MoiRNAiFold is an extension of the RNAiFold method, which utilizes the constraint programming paradigm Monte Carlo algorithm for sequence optimization (22). It finds the sequence(s) whose MFE structure is the target structure. ES + eM2dRNAs (23) uses a multiobjective evolutionary algorithm. NEMO employs a nested Monte Carlo search approach, which relies on a tree-search algorithm that explores solutions hierarchically, by recursively simulating possible outcomes and selecting the most promising paths (24). It evaluates candidate sequences using a multiobjective scoring function comprising the base pair distance and free energy difference.

## Materials and methods

### Design algorithm

DesiRNA is a new computational method for solving the RNA inverse folding problem. Solutions are scored by a multiobjective function, described in detail later in the article. Briefly, a lower score indicates a better solution. DesiRNA uses a Metropolis MC approach (25), where new candidate solutions are generated at random and then accepted or rejected based on how their scores compare to the previous solution. If a new solution is better (lower score), it is always accepted. If it is worse (higher score), the probability of accepting it depends on both the severity of the score deterioration and a parameter called the MC temperature (*T*_MC_). *T*_MC_ need not correspond to a physical temperature; it influences how easily the algorithm accepts moves that lead to poorer solutions. At low *T*_MC_ values, such moves are rarely accepted, encouraging slow, local improvements. At high *T*_MC_ values, the algorithm more frequently accepts worse solutions, allowing broader exploration of the solution space (Supplementary Figure S1).

The default variant of the algorithm implemented in DesiRNA is Replica Exchange Monte Carlo (REMC), a specific implementation of the broader parallel tempering method (26). In REMC, multiple simulations (replicas) are run in parallel at different *T*_MC_ levels. Periodically, replicas at neighboring *T*_MC_ levels attempt to exchange their configurations. If an exchange is accepted, the replicas effectively swap their *T*_MC_ levels: a replica previously running at a high *T*_MC_ will go to a lower *T*_MC_, and vice versa. In this way, a replica that found a good score at high *T*_MC_ conditions can move down to a lower *T*_MC_ level to refine its solution further. Conversely, a replica stuck at a poor solution under low *T*_MC_ conditions can be ‘heated up’ by moving to a higher *T*_MC_ level, helping it escape local minima. By dynamically moving replicas among different *T*_MC_ levels, REMC combines the global exploration facilitated at high *T*_MC_ with the local refinement possible at low *T*_MC_, making it particularly powerful for navigating complex energy landscapes, such as those encountered in RNA folding and design (Supplementary Figure S1).

The default fitness function in DesiRNA minimizes the difference between the free energy of the thermodynamic ensemble (E_pf_) and the free energy of the desired target structure (E_desired_), and can be optionally modified to include other factors in the minimization, such as the MFE, ensemble defect and the structure distance to the MFE prediction.

1. By default, DesiRNA begins the RNA sequence design process by generating a random initial sequence that adheres to the input structural and sequence constraints. This sequence generation follows the Eterna rules for RNA folding (27), providing an effective starting point for the algorithm. Alternatively, it may begin the design process with an RNA sequence provided by the user.

2. Using computational prediction, the free energy of the thermodynamic ensemble (E_pf_) and the MFE structure are calculated for each sequence [‘fc.pf()’ and ‘fc.mfe()’ functions from the ViennaRNA package (28)]. The energy of the fit of the sequence to the desired structure E_desired_ is also calculated, using the ‘fc.eval_structure()’ function, which evaluates the energy of the RNA sequence folded into the desired secondary structure. By default, a score is calculated that combines: (i) the evaluation of the difference between the E_pf_ and E_desired_ that should ideally reach the value of 0, and (ii) penalties and bonuses related to the violation or satisfaction of the user-defined restraints. Optionally, the fitness function can be extended to minimize (iii) the deviation of the MFE secondary structure from the target secondary structure according to the Matthews correlation coefficient [MCC (29) as implemented for comparison of RNA structure patterns in 1D2DSimScore (30)], (iv) the difference of E_pf_ and E_pf_ expected for a random sequence of the same length, normalized with respect to the sequence length and (v) the ensemble defect (the weighted average number of incorrect positions in the ensemble). Details of the structure prediction and energy calculation procedure are described below.

3. Multiple replicas (by default 10) of the initial sequence are created, each assigned to a distinct *T*_MC_ level, to guide the replica exchange operations. In the default settings, for levels 1–10 (corresponding to replicas 1–10), the *T*_MC_ values are equally distributed in the range of 10–150. During the REMC simulation, replicas are exchanged between different *T*_MC_ levels.

4. Each replica sequence undergoes an attempted mutation. The mutation site is chosen randomly, and the mutation is either a single nucleotide change for an unpaired nucleotide, a double mutation for two residues expected to be paired as defined by the desired structure or a set of nucleotides related by overlapping pairs in case of alternative structures. DesiRNA includes a feature that allows users to specify a percentage of attempted mutations that will target regions of the sequence not yet conforming to the correct structure (e.g., residues that are unpaired instead of paired, paired instead of unpaired or paired with an incorrect partner). This option directs mutations to these specific residues and their immediate neighbors (up to three residues away), without changing parts of the sequence already exhibiting the correct pairing pattern. In the default settings, for replicas 1–10, the fractions of targeted and completely random mutation attempts range from 70% targeted mutations in the lowest *T*_MC_ replica (to concentrate the search for improved solutions around remaining errors) to 0% in the highest *T*_MC_.

5. For each attempted mutation, the score is calculated as described in point 2. If the score of the sequence with the attempted mutation is better than the score of the original sequence in a given replica, the mutation is accepted. This new sequence, along with its score, replaces the pre-mutation sequence in the replica. However, if the score after the mutation is equal to or lower than the score before the mutation, the Metropolis criterion is applied. This criterion involves calculating a probability distribution based on the difference in scores between the mutated and original sequences, taking into account the temperature of the replica. A random number (*n*) between 0 and 1 is generated. If *p* exceeds *n*, the mutated sequence is accepted and replaces the previous sequence. Otherwise, the mutation is rejected, and the original sequence is retained. Naturally, the lower the *T*_MC_, the higher the proportion of rejected mutation attempts. To prevent sequences in low *T*_MC_ replicas from getting ‘stuck’ (without mutations) for extended periods, the mutation procedure (points 4 and 5) is attempted 100 times in each replica during one step of the MC simulation. The default parameters of DesiRNA have been set to values that, for most RNA design targets, ensure that in each step, even the sequence in the lowest *T*_MC_ replica should exhibit at least one successful mutation. Sequences in high *T*_MC_ replicas are more prone to be modified multiple times according to equation (1):


(1) \begin{eqnarray*}p\ = \min \left( {1,\exp \left[ {\frac{L}{{{{T}_{{\rm MC}}}}} \times \Delta S} \right]} \right)\end{eqnarray*}

where *p* represents the probability of accepting the move, *L* is a scaling constant or reciprocal of the Boltzmann constant, *T*_MC_ is the temperature of a given replica, and Δ*S* represents the difference in the scoring function value between the mutated and original states. The acceptance criterion ensures that moves leading to a lower energy or higher scoring function are always accepted (*P*= 1 if Δ*S*≤ 0), while moves that lead to a higher energy or lower scoring function are accepted with a probability that decreases exponentially with the size of the change and is modulated by the temperature of the system.

6. After a set number of global steps (default = 1), sequences are considered for exchange between replicas simulated at adjacent temperature levels. The goal is to reposition replicas between the *T*_MC_ levels: in general, the better she score, the higher likelihood to be moved to the low-*T*_MC_ level. This process involves pairwise comparisons of replicas, beginning with the highest *T*_MC_ and its adjacent lower *T*_MC_. The potential swap of replicas between *T*_MC_ levels is accepted or rejected by the Metropolis criterion, which considers the product of the difference in scores and the difference in the inverse *T*_MC_ values of the two replicas, according to equation (2):


(2) \begin{eqnarray*}p\ = {\rm min}\left( {1,{\rm exp}\left[ {\left( {\frac{L}{{{{T}_{{\rm MC}1}}}} - \frac{L}{{{{T}_{{\rm MC}2}}}}} \right)\ \times \ \left( {{{S}_1} - {{S}_2}} \right)} \right]} \right) \end{eqnarray*}

where *p* is the probability of accepting the exchange, *L* is a scaling constant or reciprocal of the Boltzmann constant, *T*_MC1_ and *T*_MC2_ are the temperatures of the two replicas, and *S*_1_ and *S*_2_ represent the scoring functions values of the two states to be exchanged.

7. The procedure loops back to repeat points 4–6. By default, the simulation continues for the specified number of steps or until the time of the simulation exceeds the user-defined limit. It generates RNA sequences that aim to simultaneously satisfy the goals of the scoring function described in point 2.

### Constraints and restraints

DesiRNA generates RNA sequences according to constraints provided by the user, aiming to minimize the violation of restraints. Constraints are conditions that the solution must strictly adhere to. They set hard limits on the possible outcomes and cannot be violated. In DesiRNA, constraints include:

1. Sequence length and code. The sequence must have a defined length and is specified in terms of IUPAC codes that denote different types of RNA nucleotides. Without any restrictions, the RNA sequence should be specified as a string of N characters that represent any nucleotide. The user may constrain some of the positions in the sequence to specific nucleotides A, U, C, G or their combinations, e.g., Y represents pyrimidines (C or U), S refers to G or C, etc.

2. Ability for specific residues to form base pairs. Pairwise interactions include both nested and non-nested base pairs (as in pseudoknots) within individual secondary structure patterns, and in multiple patters corresponding to alternative secondary structures. Base pairs are described in the commonly used dot-bracket notation, with paired nucleotides indicated by matching brackets (), [], {} or <> and unpaired nucleotides indicated by dots. If a pair of matching brackets is specified for any two nucleotides, only sequences are considered that are compatible with the constraint, i.e., have only G-C, C-G, A-U, U-A, G-U, or U-G at the respective two positions. If the user specifies constraints that are inherently inconsistent (e.g., that a base pair must be formed by residues at specific positions and these residues are restricted to A and G that cannot form a canonical pair), DesiRNA will inform the user that a solution that satisfies the constraints cannot be obtained.

Restraints are the desired parameters of the solution, and the minimization of the violation of restraints is an element of the scoring function, which steers the sequence design in a certain direction. Violation of restraints reduces the score of a solution but does not prevent DesiRNA from considering that solution. DesiRNA can optimize the conformance of the solution to the restraints, even if they cannot be fully satisfied. Restraints in DesiRNA include:

1. ACGU content, i.e., the relative fraction of the four nucleotide residues.

2. The presence or absence of specific sequence motifs.

3. Ability to form homodimers, heterodimers and homooligomers.

4. Negative design, i.e., minimizing the chance of the RNA sequence folding into structures other than the target structure.

In DesiRNA, constraints must be strictly fulfilled, and sequences are generated to automatically satisfy them, whereas restraints represent preferences that influence the scoring and guide the optimization process but do not necessarily need to be fully satisfied. For example, constraining the sequence to include G residues at 60% of specific positions while simultaneously restraining it to minimize the percentage of G residues to zero creates an inherent conflict. This situation enforces the constraints strictly, allowing only limited fulfillment of the restraints within the bounds set by the constraints. It should be emphasized that while the chemical compatibility to form a base pair by residues at two specified position is a constraint (must be fulfilled, sequences without such a compatibility are not generated), the actual formation of this base pair in the predicted secondary structure is a restraint (a violation leads to a penalty, but sequences that do not have the ideal secondary structure are generated during the design process).

### DesiRNA output

During the simulation, DesiRNA generates trajectories of RNA sequences with accepted mutations, along with their predicted structures and scores. Once the simulation is complete, DesiRNA performs standard post-processing to analyze the outcomes and present them to the user. This involves evaluating the predicted structures of the RNA sequences against the target structure and quantifying their similarity using MCC (see above) to measure how accurately the predicted base pairs match the target structure, with a score of 1 indicating a perfect match and 0 indicating no correlation. Since DesiRNA is focused on the minimization of the fitness function, we use 1-MCC, where zero corresponds to a perfect match, the results can be sorted easily to highlight the best solutions. The DesiRNA output file is a CSV-formatted table listing the best-scoring sequences (10 by default). Each row indicates the secondary structure, the corresponding sequence, its E_pf_, a relative score of the fitness function (the lower the better), and information about fulfilling optional constraints and/or restraints.

### Secondary structure prediction and energy evaluation

In step 2, RNA secondary structure prediction and energy evaluation require a method that can be integrated into the Python framework to calculate both the E_pf_ for an RNA sequence and E_desired_ for that sequence versus the desired structure. There are a limited number of such methods, including ViennaRNA (28), RNAstructure (31) and NUPACK (32). Because of superior performance in terms of speed, we chose to use the ViennaRNA package with the Turner energy model, available in either 1999 (33) or 2004 (34) versions. ViennaRNA is also frequently used for benchmarking RNA design methods, enabling us to directly compare DesiRNA performance with published results from other methods on the Eterna100 benchmark. Specifically, the ‘fc.pf()’ function from ViennaRNA calculates the free energy of the thermodynamic ensemble of an RNA sequence (partition function), while the ‘fc.eval_structure()’ function determines the energy of an RNA sequence folded into the desired secondary structure.

### Design of pseudoknotted sequences

One limitation of the standard algorithms provided by the ViennaRNA package is their inability to predict pseudoknots or to assess the energy of folding into a pseudoknotted structure. DesiRNA allows the desired structure to contain pseudoknots, and to enable pseudoknot prediction for candidate RNA sequences, we implemented a stepwise procedure. Following the initial prediction of nested pairs, we encode them as x-type restraints that prevent base-pairing and rerun RNAfold to predict extra pairs only among the nucleotides that were unpaired in the previous iteration. This procedure is iterated the number of times corresponding to the depth of pseudoknots in the desired structure unless no additional pairs are predicted in a given iteration. The sets of base pairs predicted in subsequent iterations are combined to form a pseudoknotted predicted structure, which is then compared to the desired pseudoknotted structure as described in point 2. However, due to the inability of RNAfold to predict E_pf_ and E_desired_ for pseudoknotted structures, the energy comparison is limited to the first level of prediction.

An alternative approach that supports the consideration of pseudoknots may involve the RNAstructure package, which is capable of calculating both E_pf_ and E_desired_ for pseudoknotted structures. However, we found the available implementation of RNAstructure too slow for DesiRNA (∼100 times slower). Alternative methods able to predict pseudoknots that we tested and confirmed to work with DesiRNA to calculate E_pf_ and/or MFE include HotKnots, DotKnot and IPknot. However, we were unable to implement these methods to calculate E_desired_ in the way that is essential for the DesiRNA scoring function.

### Design of sequences compatible with alternative structures

DesiRNA enables the design of RNA sequences that can fold into multiple alternative structures. The program enables users to specify an arbitrary number of desired secondary structures within the input file. The assessment of the compatibility of these structures, to determine if a sequence fulfilling all specified conformations is feasible, is done using the graph coloring approach (35). This method assigns ‘colors’ to the vertices of a graph such that no two adjacent vertices share the same color. A graph is composed of nodes (vertices) and edges, where edges represent relationships or constraints between nodes. The algorithm assigns colors iteratively, ensuring that no conflicting assignments occur along an edge. This approach is widely applied to constraint satisfaction problems where relationships between variables must be respected. In DesiRNA, RNA secondary structures are represented as graphs, where each nucleotide is a vertex and base-pairing interactions are edges. The graph coloring algorithm is adapted to assign nucleotide identities (colors) to vertices while satisfying base-pairing rules (e.g., A-U, G-C, G-U). For multiple target structures, the algorithm ensures that the same sequence (vertex assignments) can fulfill the base-pairing requirements across all specified structures. The compatibility check performed by this method identifies conflicts or verifies feasibility before proceeding to the sequence optimization phase.

Following this preliminary evaluation, DesiRNA calculates E_desired_ of a sequence for each of the structures specified by the user. The objective is to minimize the energy difference between E_desired_ for each input structure and E_pf_, optimizing the sequence to potentially fold into each of the proposed alternative structures. This methodology allows for the design of sequences that are able to fold into distinct structural states with comparably low energies, suitable for applications in creating riboswitches and other complex RNA molecules.

### Application of negative design

DesiRNA offers an option for the negative design, which aims to minimize the chance of the RNA sequence folding into structures other than the target structure. Specifically, the scoring function is adjusted to penalize sequences that can fold into low-energy suboptimal structures while rewarding sequences that maximize the energy difference between the desired structure and the most stable undesired structure. This procedure aims to maximize the energy gap between the desired and undesired structures, reducing the probability of the RNA folding into competing conformations. When this option is activated and the E_pf_ structure matches the target structure, an adjustment to the scoring function is made to increase the energy difference between the E_pf_ structure and the next best suboptimal structure. This process is computationally intense, so it is not activated by default. It is advised to use this option for further optimization of sequences that already match the desired design.

### Specifying nucleotide composition preferences

Due to the energy model’s preferences, designed RNA sequences can contain long stretches of a single nucleotide type or only have GC pairs in the helices. To address this in DesiRNA, the ‘*-acgu on*’ option can guide the design process to produce sequences with the user-defined nucleotide composition. The default nucleotide content for this option is A:15%, C:35%, G:35%, U:15%, and it can be modified by the user.

### Design of RNA–RNA complexes

DesiRNA allows the design of RNA–RNA complexes by incorporating sequence and structural constraints for two interacting molecules. To do this, users should input two sequences separated by the ‘&’ symbol, e.g., ‘NNNNNN&NNNNNNNN’ and provide the structure of the dimer in the dot-bracket notation, e.g., ‘((((..&….))))’. Both homo- and hetero- dimers can be designed and both symmetric and asymmetric secondary structures are accepted. In the design of RNA-complexes the scoring function is chosen that maximizes the desired oligomeric state in the solution, i.e., the product of two molar fractions: the fraction of complexes that assume the desired structure and the fraction of the RNA molecules that form a dimer as opposed to monomers.

### Control of oligomerization

The user can specify that in the design of single-chain structures, the algorithm will give preference to RNA sequences that would form monomers instead of oligomeric complexes. In this case the fitness function would maximize the fraction of RNA molecules in the thermodynamic ensemble that would be of the desired structure (monomeric versus oligomeric). The default fitness function optimized in DesiRNA is E_pf_(seq) – E_desired_(seq):


(3) \begin{eqnarray*}kT{\mathrm{log}}[{{{\mathrm{x}}}_{{\mathrm{desired}}}}\left( {{\mathrm{seq}}} \right)]{\mathrm{\ }} = {\mathrm{\ }}{{{\mathrm{E}}}_{{\mathrm{pf}}}}\left( {{\mathrm{seq}}} \right){\mathrm{\ }} - {{{\mathrm{E}}}_{{\mathrm{desired}}}}\left( {{\mathrm{seq}}} \right)\end{eqnarray*}

which is related to x_desired_(seq) — the fraction of RNA molecules in the thermodynamic ensemble that assume the desired structure, provided only single-chain structures are allowed to be formed. To prevent oligomerization, what is being maximized is the product of x_desired_(seq), i.e., the probability that a single RNA chain assumes the desired secondary structure, and of x_monomer_(seq), i.e., the probability that in solution the given RNA chain stays monomeric.

The x_monomer_(seq) fraction can itself be computed by considering the equilibrium between monomeric and dimeric states, as analyzed in (32). As the default fitness function is proportional to log[x_desired_(seq)], the use of the product of x_desired_(seq) and x_monomer_(seq) instead of x_desired_(seq) corresponds to including an additive correction proportional to log[x_desired_(seq)]. If this option is enabled by the user, the fitness function is modified depending on whether monomeric or dimeric RNA structures are desired in the thermodynamic ensemble of the designed sequence. The additional term involves the computed x_monomer_(seq) and reads:


(4) \begin{eqnarray*}{{{\mathrm{E}}}_{{\mathrm{pf}}}}({{\mathrm{seq}}} ) - {{{\mathrm{E}}}_{{\mathrm{desired}}}}( {{\mathrm{seq}}} )+{kT}\,{\mathrm{log}}[{{{\mathrm{x}}}_{{\mathrm{monomer}}}}( {{\mathrm{seq}}} )]\end{eqnarray*}

for monomeric sequence design, and:


(5) \begin{eqnarray*}{{{\mathrm{E}}}_{{\mathrm{pf}}}}( {{\mathrm{seq}}} ) - {{{\mathrm{E}}}_{{\mathrm{desired}}}}( {{\mathrm{seq}}} )+{kT}\,{\mathrm{log}}[1 - {{{\mathrm{x}}}_{{\mathrm{monomer}}}}( {{\mathrm{seq}}} )]\end{eqnarray*}

for dimer sequence design.

To evaluate the propensity of the designed sequences to form a monomer versus a dimer the monomer formation percentage (MFP) and the dimer formation percentage (DFP) is used. The MFP evaluates the likelihood that a sequence will form a monomer and is calculated as the molar fraction of monomeric molecules x_monomer_(seq) multiplied by 100% [MFP = x_monomer_(seq) × 100%]. The DFP evaluates the likelihood that a sequence will form an oligomeric structure and is calculated as the complement of the molar fraction of monomeric molecules [1 − x_monomer_(seq)] multiplied by 100% [DFP = (1 − x_monomer_(seq)) × 100%].

### RNA synthesis and purification

The DNA templates were purchased as double-stranded DNA strings (Gene strings, Thermo Fisher Scientific Inc.), which are PCR-amplified to incorporate T7 promoter sequence (Supplementary Table S1). The *in vitro* transcription (IVT) reactions were performed using our in-house T7 RNA polymerase (0.6 U/μl) with a custom reaction mixture containing 1.5 mM NTPs, 50 ng/μl DNA template in 1× IVT buffer (40 mM Tris, pH 8.0, 10 mM DTT, 2 mM spermidine and 0.01% TritonX-100). Following IVT, the transcribed RNAs were treated with DNase I and resolved the RNAs on an 8% (w/v) polyacrylamide gel containing 8 M urea. The corresponding bands of RNA were excised from the gel and eluted in 100 mM Tris–HCl (pH 6.5), 250 mM NaCl and 10 mM EDTA. The RNA samples were further concentrated using the ethanol precipitation method, the RNA pellets were resuspended in nuclease-free water and stored at −80°C prior to subsequent experimental analysis.

### Ribozyme cleavage assays

To assess the *cis-*cleavage activity of the *glmS* design RNAs, we performed IVT reactions in the presence and absence of 2 mM GlcN6P. Following DNase I treatment, the IVT products were denatured in formamide and separated on an 8% (w/v) polyacrylamide gel containing 8 M urea. The gel was stained with ethidium bromide staining and visualized using GelDoc (Bio-Rad).

The *glmS* design RNAs trans-cleavage assay was performed with 1:1.5 ratio of Cy5-labeled RNA substrates and with their respective *glmS*_WT, *glmS*_D1 RNA enzymes (Supplementary Table S2). The substrates and enzyme RNAs were separately dissolved in nuclease-free water and denatured at 95°C for 2 min, rapidly cooled on ice for 2 min. The RNAs were mixed in 1× the activity buffer composed of 50 mM Hepes (pH 7.5), 100 mM KCl, and 10 mM MgCl2 and the sample was incubated at 37°C for 15 min. The folded RNA mixture was then incubated at room temperature for 5 min. For the zero time-point of the trans-cleavage activity assay, 10 μl of the preincubation was removed. The RNA trans-cleavage reactions were initiated by adding 1 mM GlN6P. Aliquots of the reactions were taken at different time points (5, 15 and 30 min), and the reactions were terminated by adding an equal volume of formamide containing 100 mM EDTA. The samples were heated to 95°C for 2 min, separated on a 20% (w/v) polyacrylamide gel containing 8 M urea and visualized using the ChemicDoc imaging system (Bio-Rad). The percentages of the cleaved products were quantified with the ImageJ software (36).

### Hardware configuration for RNA design simulations

For the 1 min simulations, we used a laptop equipped with an AMD Ryzen 9 5900HX processor (3.3 GHz, 16 threads) and 32 GB of RAM, running Ubuntu 18.04 and Python 3.6. For the 1 and 24 h simulations conducted to solve the remaining EteRNA100 puzzles, we used a computational cluster running Ubuntu 22.04.1 LTS. The cluster consisted of five nodes, each equipped with Intel(R) Xeon(R) Gold 6126 CPUs (2.60 GHz, 48 cores) and 128 GB of RAM. Each simulation run utilized 10 threads, ensuring efficient parallel execution of the REMC simulations. We used this hardware configuration for the full benchmark of DesiRNA and additional tests on top-performing methods available as standalone implementations, specifically those reporting at least 75/100 successful designs within 24 h. For NEMO (95/100), ES + eM2dRNAs (94/100), SentRNA (78/100) and SAMFEO (77/100), we tested performance on designs they failed to solve in 24 h. MoiRNAiFold (91/100) was only accessible via a web server and could not be tested under our setup.

### 3D folding simulations with SimRNA

To validate the 3D structures formed by RNA sequences designed by DesiRNA, we used the SimRNA (37,38) method, which uses a coarse-grained representation and a statistical potential to carry out MC dynamics simulations. SimRNA has been validated in benchmarks of RNA 3D structure prediction including RNA-Puzzles (39,40) and CASP (41,42) consistently ranking among the top-performing methods. SimRNA was run with default parameters. Any modifications of the standard workflow used for data processing are indicated in the ‘Results’ section. Integrating DesiRNA with SimRNA improves the validation of RNA sequences. DesiRNA focuses on generating sequences for specific secondary structures, while SimRNA evaluates these sequences in a 3D context to assess their structural configurations. This approach allows for the computational assessment of the folding behavior. Early-stage 3D validation with SimRNA provides additional confidence in the stability and functionality of designed RNAs, complementing the secondary structure design process.

### Availability of software

DesiRNA is freely available on GitHub (https://github.com/fryzjergda/DesiRNA.git). The version submitted for publication has been included as a Supplementary File. The software is implemented in Python 3.6 and includes a comprehensive installation manual providing detailed instructions for setting up and configuring all required libraries and dependencies. DesiRNA installation was tested on Ubuntu 20.04 and 22.04, MacOS, Python versions 3.6, 3.7, 3.8, 3.9 and the program supports installation via Conda.

## Results

### Evaluation of DesiRNA performance on the Eterna benchmark

Our primary aim was to compare DesiRNA performance with previous approaches in addressing complex and established RNA design challenges. The Eterna100 benchmark offers a set of 100 RNA secondary structures, many of which pose significant challenges for sequence design due to lone base pairs, large internal loops and intricate multiloops. The standard in the field is to compare performance using the original version of the Eterna100 benchmark (V1), compatible with the Turner1999 (33) but not the Turner2004 (34) energy rules. In particular, testing the ability to solve each Eterna100 puzzle in 24 h serves as a widely accepted timeframe for assessing computational RNA design tools (21). According to the criteria commonly used for testing RNA design methods, a puzzle is considered ‘solved’ if the predicted structure of a designed sequence matches the target structure exactly, with even a single base pair difference rendering the design ‘unsolved’.

To date, none of the existing RNA design methods has been able to solve all 100 puzzles as documented in the respective publications (22,24,32,43–54). The state-of-the-art was represented by NEMO (24), which solved 95 of the Eterna100 V1 puzzles within a 24 h limit. When DesiRNA was run with default parameters, using the basic energy function set to the Turner1999 model, it solved 90 of these puzzles in under 1 min, an additional five in under 1 h, and all Eterna100 puzzles within 24 h (Supplementary Figures S2 and S3). To ensure a fair comparison with other tools, we used our computational setup (see ‘Materials and methods’ section for details) to test other methods that had previously reported at least 75/100 successful designs within 24 h. Among the methods tested, we achieved only three additional successful designs with SAMFEO (Supplementary Figure S4).

**Figure 1.**
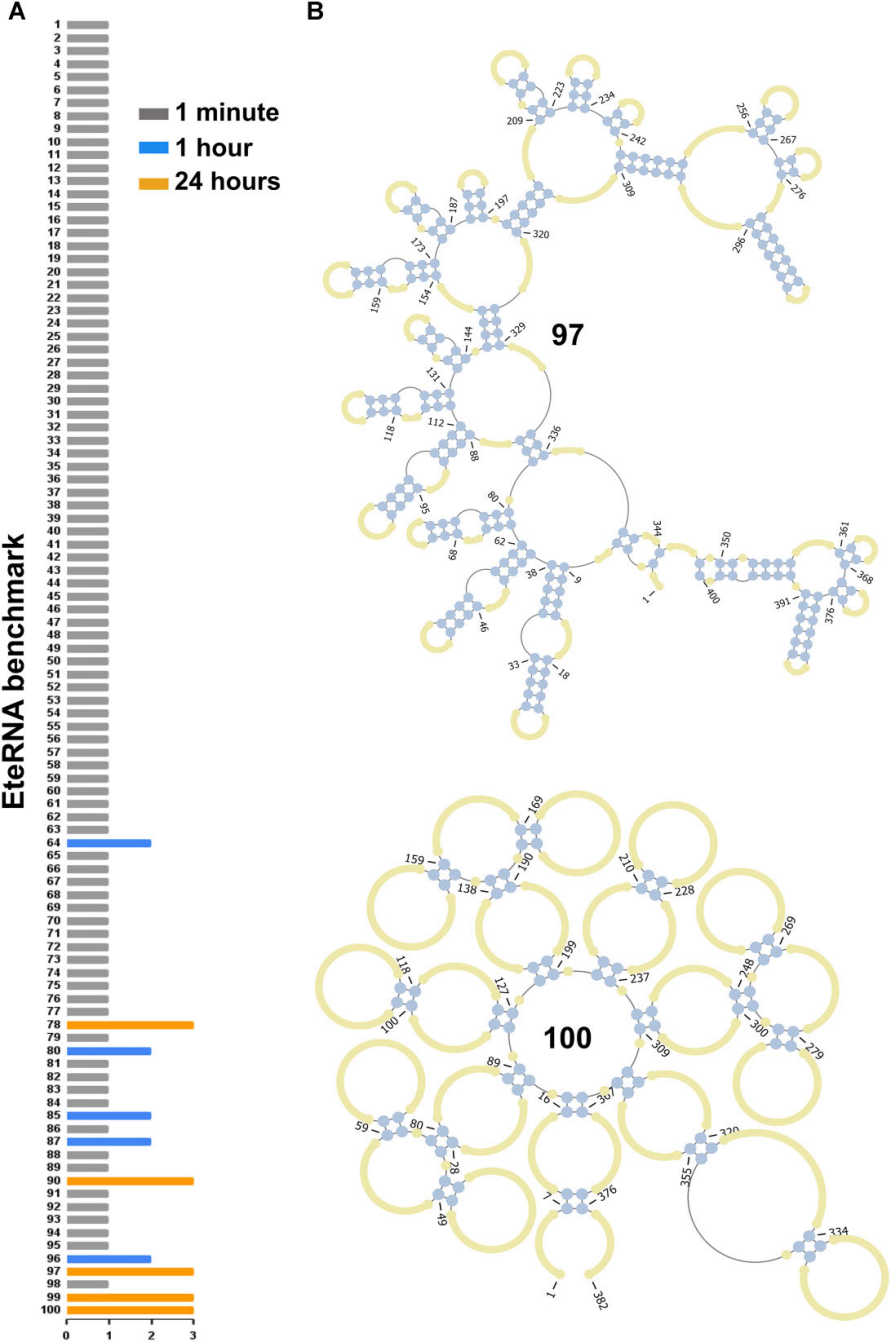
DesiRNA performance on the EteRNA100 benchmark dataset. (**A**) The EteRNA100 V1 dataset versus time points (1 min, 1 h and 24 h). Ninety of these puzzles were solved within 1 min, puzzles 64, 80, 85, 87 and 96 were solved within 1 h, and the remaining puzzles were solved within 24 h. (**B**) DesiRNA solved RNA puzzles (97 and 100) that were not solved within 24 h by any method to date.

Supplementary Table S3 presents the designed sequences and Figure 1 presents the summary of the benchmark with the two structures solved automatically for the first time by DesiRNA. We also tested the performance of DesiRNA on the Eterna 100 V2 benchmark (55) redesigned for the compatibility with the Turner2004 model, using the Turner2004 model accordingly. Like for the Turner1999 model, the results were better than reported previously for any other automated method (Supplementary Table S4). With the Turner2004 model, DesiRNA solved 97 out of 100 puzzles within 24 hours (85 under a minute, 95 under 1 h).

### Example design of a ribozyme, with experimental validation

For the experimental validation of DesiRNA design sequences, we selected the *glmS* ribozyme, a catalytic RNA molecule that exhibits self-cleaving activity upon binding to the cofactor glucosamine-6-phosphate(GlcN6P) (56,57). The 3D structure of the *glmS* ribozyme from *Thermoanaerobacter tengcongensis* has been determined by X-ray crystallography (PDB: 2Z75)(57,58). In this work we will refer to this ribozyme variant as *glmS*_WT. The secondary structure comprises four helices (P1–P4), with the core catalytic region formed by a double pseudoknot that positions the central helix P2.1, cradling the scissile phosphate in its major groove. The adjacent helix P2.2 carries the scissile phosphate at its 5′ end and engages in metabolite binding through its major groove (58,59). Constraints on conserved residues (at least 97% identity) were generated based on the Rfam RF00234 family sequence alignment (60), and the base-pairing constraints were generated based on the RF00234 consensus structure as well as the secondary structure of *glmS*_WT.

**Figure 2.**
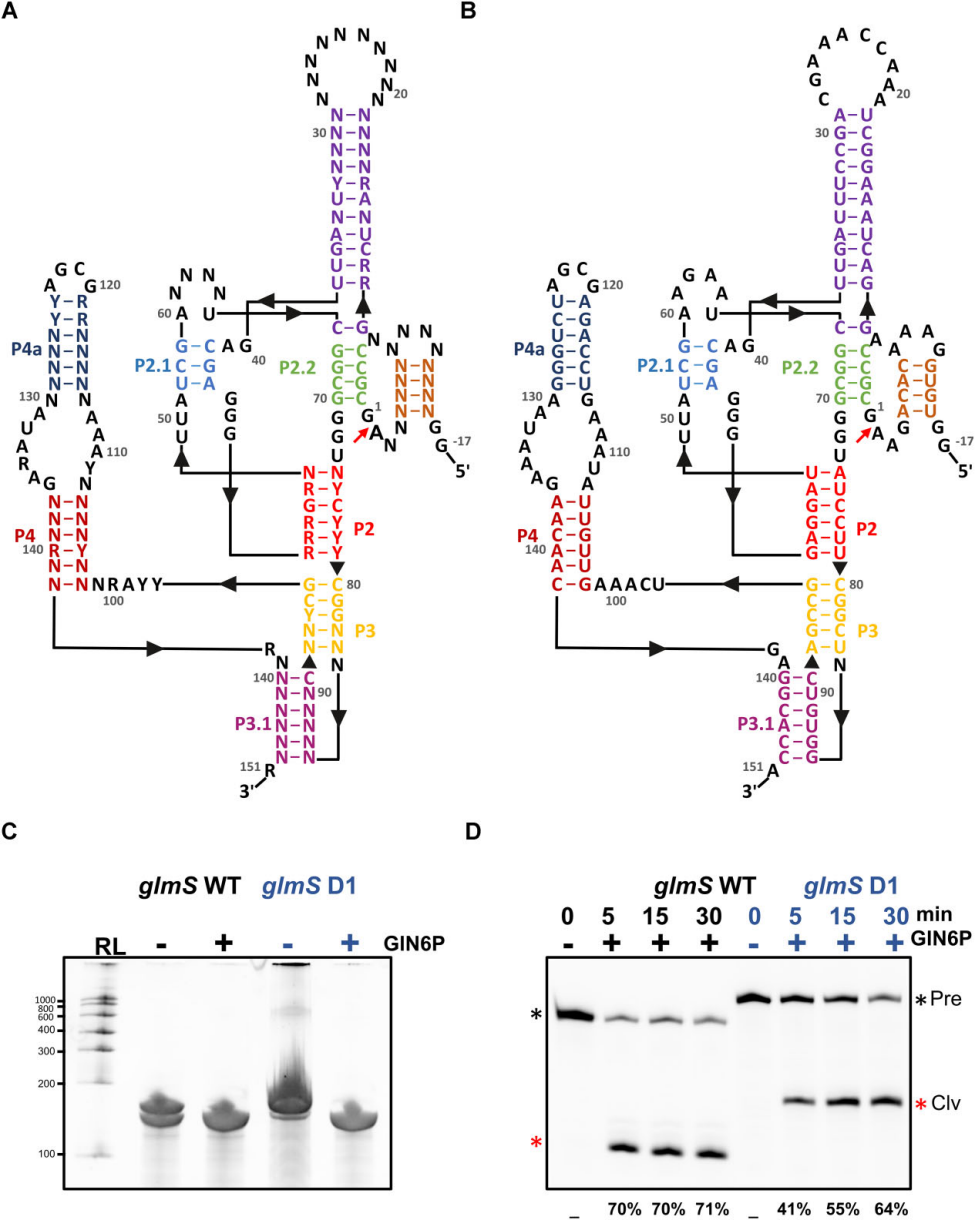
*glmS* ribozyme sequences design and experimental validation. (**A**) *glmS* design constraints were based on the RF00234 family consensus sequence, and we utilized the crystal structure of *T. tengcongensis glmS* ribozyme (PDB ID: 2Z75) as a reference. (**B**) *glmS*_D1 sequence designed with DesiRNA. (**C**) Cis-cleavage activity, IVT in the absence and presence of 2 mM GlN6P. The denaturing gel analysis of the IVT products shows the transcription of shorter RNA products in the presence of GlN6P. (**D**) In trans-cleavage activity, Cy5-labelled substrates were used along with the catalytic part of the respective designs and *glmS*_WT RNAs. Reaction products were collected at time points 0, 5, 15, and 30 min, and the samples were analyzed on a denaturing gel. The substrate (Pre, black) and product (Clv, red) bands are indicated with asterisks.

DesiRNA was used to design *glmS* RNA sequence variants, using a standard protocol, with constraints mentioned above. For top-scored designs (Supplementary Table S5), we conducted additional secondary structure prediction with various methods, and selected three sequences, for which predictions agreed best with the desired base-pairing pattern. The three top-scored sequences, *glmS*_D1, *glmS*_D2, and *glmS*_D3 were subjected to experimental validation in a self-cleavage assay (see ‘Materials and methods’ section). In IVT reaction in the absence of GlN6P, *glmS*_WT, *glmS*_D2, and *glmS*_D3 displayed partial self-cleavage activity (Supplementary Figure S5A), while the *glmS*_D1 design RNA showed more pronounced formation of a full-length RNA, indicating minimal self-cleavage activity (Figure 2). Upon addition of 2 mM GlN6P in IVT reactions, we observed shortened RNA transcription products for both *glmS*_WT and *glmS* design RNAs. Additionally, we conducted *in vitro* trans-cleavage activity assays using Cy5-labeled RNA substrates for the respective *glmS*_WT and *glmS* design RNAs (Supplementary Table S2). The maximum efficiency of cleaved product formation was 71% for *glmS*_WT, whereas *glmS*_D1 exhibited 64% (Figure 2D), *glmS*_D2 and *glmS*_D3 showed ∼80% efficiency (Supplementary Figure S5B) in 30 min of the reactions. These results indicate that *glmS* design RNAs undergo both *cis* and trans cleavage in the presence of GlN6P, with efficiencies close to those of *glmS*_WT RNA (Supplementary Figure S5).

### Design of RNAs with the same sequence motifs, and a propensity to form either monomeric or homodimeric structures

To evaluate DesiRNA’s ability to design RNA sequences for monomeric versus dimeric states, we selected a 19 nt motif from the bacteriophage Mu RNA molecule, which binds to the Com protein. This RNA motif is known to fold into a monomeric hairpin but can also dimerize into a homodimeric duplex. Previously, using a very early version of DesiRNA, we designed monomeric (RNA-I) and dimeric (RNA-II) versions of the Com-binding RNA (61). The design simulations focused on preserving the consensus motif recognized by the Com protein, specifically a bipartite motif comprising two instances of the sequence 5′-GAD(N)_2_HC-3′, as identified by *in vitro* selection experiments. We experimentally validated that RNA-I exclusively formed a monomer, while RNA-II was predominantly dimeric, with a minor monomeric fraction. We determined that the Com protein bound much better to RNA-I than to RNA-II, indicating that the monomeric form is most likely the biologically relevant one. We also determined a crystal structure of RNA-II, which formed a dimer in the crystal (61).

**Figure 3.**
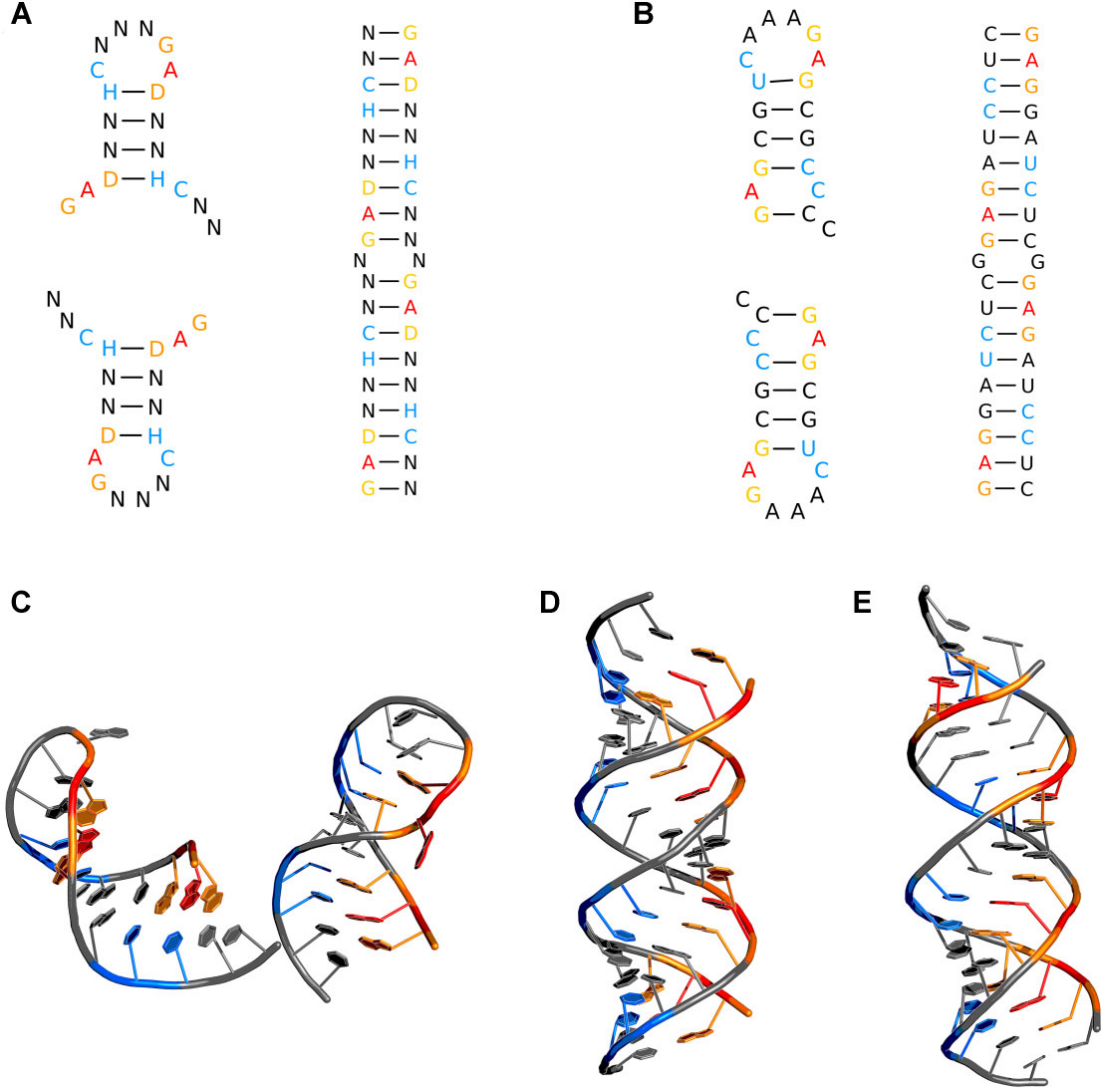
Mom19-I and Mom19-II sequence design. (**A**) Mom19-I and Mom19-II sequence design constraints. (**B**) Sequence and secondary structure of the designed Mom19-I and Mom19-II (**C**) 3D representative of the prevalent structure observed in SimRNA folding simulations for Mom19-I. (**D**) 3D representative of the prevalent structure observed in SimRNA folding simulations for Mom19-II. (**E**) Crystal structure of RNA-II structure (RCSB PDB ID: 6IA2). The colored residues represent the bipartite 5′-GAD(N)_2_HC-3′ motif.

For testing the current version of DesiRNA, we designed two Com-binding 19-mer RNA sequence variants with a propensity to form a monomer (Mom19-I) or a homodimer (Mom19-II), respectively. As previously, the consensus motif, essential for Com protein interaction, was constrained throughout the design process (Figure 3A). Sequence constraints for both designs were specified as ‘GADNNHCNNNGAGNN&GADNNHCNNNGAGNN’ incorporating the bipartite 5′-GAD(N)_2_HC-3′ motif (61). Base-pairing constraints were set to ‘..((((……))))…&..((((……))))…’ for the monomer design and ‘(((((((((.(((((((((&))))))))).)))))))))’ for the dimer design. The simulation was run with the ‘*-d on*’ option (the dimer design mode). The simulation time was set to 60 s using the ‘*-t 60*’ flag. Sequences and their features are presented in Supplementary Table S6 and the structures of the best variants are shown in Figure 3.

To evaluate the propensity of the designed sequences to form a monomer versus a dimer, the monomer (MFP) versus DFP values were calculated, respectively (see ‘Materials and methods’ section—Prevention of oligomerization). The calculated monomeric fraction for Mom19-I was 99.972%, while the dimeric fraction for Mom19-II was 99.384%, indicating the desired preference was fulfilled by the designed sequences. The respective measures of previously designed RNAs were 99.956% for RNA-I (monomer) and 99.084% for RNA-II (dimer), indicating consistently high performance of DesiRNA.

For additional validation of the oligomerization preferences of Mom19-I and Mom19-II, we conducted computational 3D folding simulations using SimRNA (37,38), starting with the monomeric unfolded forms of these RNAs. The goal of the simulations was to test their tendency to maintain the monomeric structure or to refold into a dimer. For Mom19-I simulation, 98.675% of best-scored structures (10% of frames) in the lowest temperature level remained as monomers. For Mom19-II, 100% of 10% best-scored structures in the lowest temperature level were dimers, indicating that the designed sequence drove the monomers to unfold and refold as dimers. Summarizing, with DesiRNA we were able to design sequences that retain common sequence motifs defined by constraints, while the remaining sequence features drive them to fold into two different oligomeric states.

### Design of a single RNA sequence capable of folding into two alternative structures

**Figure 4.**
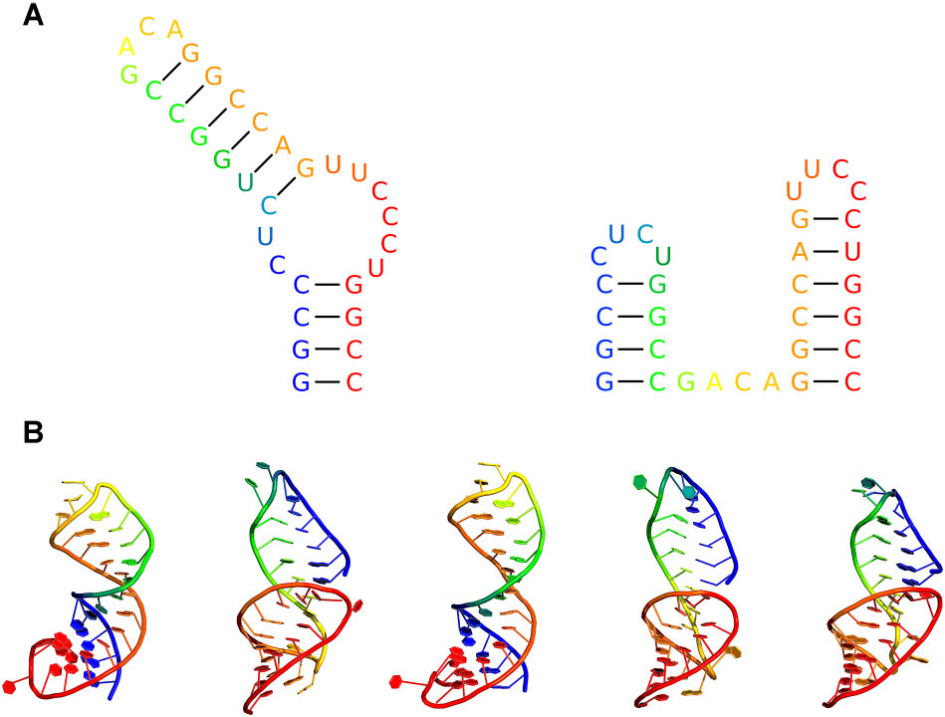
DesiRNA-designed RNA sequence with a comparable propensity to fold into two very different secondary structures. (**A**) One sequence and two secondary structures of the designed RNA: a helical stem with a double loop and two separate hairpins. (**B**) 3D models representing the largest five clusters generated by SimRNA simulations for the top designed solution. First and third clusters exhibit the helical stem conformation, while the second, fourth and fifth clusters exhibit the two-hairpin conformation.

To assess DesiRNA functionality in designing RNA sequences capable of adopting different secondary structure patterns, we challenged it to design one sequence that could fold into two forms: a helical stem with a double loop or two separate hairpins (Figure 4A, Supplementary Table S6). No sequence constraints were used, and the base-pairing constraints for two alternative structures were defined as ‘((((..((((((….))))))……))))’ and ‘((((….))))….((((((….))))))’. The alternative structure design scheme was activated automatically, as always with two or more structural constraints in the input file. The simulation time was set to 60 s using the ‘*-t 60*’ option, and the energy model parameter was specified with the ‘*-p 2004*’ flag.

For the best-scored sequence (Figure 4), ViennaRNA-calculated energies of the two alternative structures were −16.8 kcal/mol versus −16.7 kcal/mol, respectively, indicating a comparable preference for the designed sequence to adopt one of the two desired structures. Further validation was conducted through SimRNA simulations to determine the sequence’s ability to fold in 3D, while accommodating either of the predefined secondary structures. Analysis of 1% best-scored SimRNA-generated conformations revealed that the two largest clusters accounted for 61% and 21% of the conformations in the one-hairpin versus two-hairpins configurations, respectively. Similarly, when 5% of the best-scored models were analyzed, the two largest clusters corresponded to 52% and 22% of the conformations in the one-hairpin versus two-hairpins configurations, respectively. These results support the capability of DesiRNA to design sequences capable of adopting very different structures with comparable energies and frequencies of occurrence in folding simulations.

### Application of the negative design

In this example, the negative design principle was applied to design an RNA sequence that preferentially folds into a specific structure while avoiding alternative structures as much as possible. The objective was to significantly lower the energy of the target structure compared to suboptimal alternatives. For testing, we selected the signal recognition particle (SRP) RNA, 136 nt long, due to its relatively complex folding pattern, including a three-way junction and several bulges. We chose an SRP variant from *Methanocaldococcus jannaschii*, for which a crystal structure is available (3NDB) (62).

Sequence constraints were derived from the Rfam conserved sequence for Archaeal SRP RNA (RF01857) family (63) in the form ‘NNNNNGNCCNNNNNNNNNNNNNGNNNNNNNNNNNNNNNNNNNAGGNNNNNNNNNNNNNNNNNNNNNNNNNNNANNNNNNNAGGCNCGGAAGNGAGCANNNNNNNNNNNNNCNNNNNNNNNNNNNGGNNNNCNNNNN’. The base-pairing constraints were based on the crystal structure (3NDB)(62) as ‘(((((((((((((((((.(((((..(((((.(((((((((….)))))))))..)))))….((((((…..(((…..(((….)))…..)))..)))))).))))))).))))))))…)))))))’. The simulation time was set to 600 s (‘*-t 600*’) to allow comprehensive exploration of the sequence space; the negative design scheme was activated (‘*-nd on*’) to prioritize the formation of the target structure while minimizing the likelihood of forming alternative conformations; the oligomerization prevention was turned on to ensure that the designed RNA sequence remained monomeric (‘*-o on*’); the ‘*-acgu on*’ was used to maintain a natural composition of ACGU nucleotides; the Turner 2004 energy model was used (‘*-p 2004*’).

Results of this design are presented in Supplementary Table S6. ViennaRNA RNAfold correctly predicted the secondary structure of *M. jannaschii* SRP, with the free energy of the thermodynamic ensemble of −78.82 kcal/mol, the MFE structure energy of −77.60 kcal/mol, frequency of the MFE structure in the ensemble at 13.79% and the ensemble diversity of 13.75. In comparison, the designed SRP-like sequence (Supplementary Table S6) was also able to form the target structure, had the free energy of the thermodynamic ensemble of −82.22 kcal/mol, MFE energy of −82.10 kcal/mol (lower than for the native sequence), a significantly increased MFE structure frequency of 80.18% and a greatly reduced ensemble diversity of 1.06. This indicates the effective application of negative design principles in DesiRNA, improving sequence specificity and stability for a specified conformation.

## Discussion

The design of RNA molecules that fold into specific structures and exhibit desired functionalities is an exciting area in computational biology. Custom-designed RNA molecules have potential applications in therapeutics, diagnostics, synthetic biology, nanotechnology and as RNA-based sensors. For instance, in therapeutics and diagnostics, RNA molecules can target specific cellular pathways or act as RNA-based drugs. In synthetic biology, they can regulate gene expression, and in nanotechnology, their intricate 3D structures can be harnessed for nano-scale devices.

To address the challenges of RNA sequence design, we developed DesiRNA, a method to design RNA sequences that fold into specific desired structures while accommodating a set of user-defined constraints and restraints. DesiRNA was specifically designed to support the design of sequences that fold into desired secondary as well as 3D structures, contain features typical of functional RNAs such as pseudoknots, account for oligomerization and retain the sequence composition typical of natural RNAs. An important feature of DesiRNA is the ability to design RNA sequences that can fold into multiple structures, allowing the design of RNAs that are able to switch between functional states in response to environmental cues. DesiRNA can be also used to find sequence variants that minimize the formation of alternative structures.

We subjected DesiRNA to comparison against other RNA design algorithms using the Eterna100 benchmark. DesiRNA solved 90 puzzles in a 1 min run, a number that increased to 95 in an hour, and to all 100 in a 24 h period. DesiRNA is thus the first method to solve the whole Eterna100 V1 benchmark within a 24 h time limit. Previously, the state-of-the-art was represented by NEMO, which solved 95 puzzles within that time limit. In addition to the Eterna100 dataset, which contains mostly artificial structures, we used DesiRNA to redesign the naturally occurring *glmS* ribozyme sequence, with minimal sequence constraints to achieve the desired complicated secondary structure with a double pseudoknot. We validated experimentally the functionality of the selected designed *glmS* sequences. These design sequences exhibited an efficiency of self-cleavage activities comparable to that of the reference *glmS* ribozyme. We also provided examples of successful RNA sequence design challenges focused on specific features: design of monomeric versus dimeric forms, capability to fold into two alternative structures, and the use of negative design.

Due to the stochastic nature of the design process in DesiRNA, MC simulations do not guarantee the identification of the optimal solution. For challenging design targets that do not yield satisfactory results on the initial attempt, it is recommended to conduct multiple independent simulations—by default 10, each initiated with a different random seed. Additionally, starting this process with various RNA sequences can significantly enhance the exploration of the solution space. The relationship between the seed sequence and the resulting designs depends on multiple factors. While our tests showed that, for long simulations, the sequence composition of designed RNAs is often similar regardless of the starting sequence, RNA length, simulation duration, the range of *T*_MC_ in the REMC simulation, and the use of constraints and restraints significantly impact the exploration of sequence space. This highlights the importance of carefully selecting parameters for specific applications. Users may adjust parameters based on their specific needs and the nature of the design problem, including: (i) the number of replicas, (ii) the MC temperature range and (iii) the fraction of targeted mutations. DesiRNA can be run iteratively, using suboptimal solutions from previous runs as new starting points, especially sequences that meet the design criteria only partially. With its default settings, DesiRNA surpasses the current state of the art in fully automated RNA sequence design and provides a versatile framework to further enhance solution space sampling for even the most challenging RNA sequence design problems.

## Supplementary Material

gkae1306_Supplemental_Files

## Data Availability

The data underlying this article are available in the article and in its online supplementary material. DesiRNA is freely available on GitHub (https://github.com/fryzjergda/DesiRNA.git). The version submitted for publication has been included as a Supplementary File.

## Notes

Present address: Farhang Jaryani, Human Genome Sequencing Center, Baylor College of Medicine, Houston, TX 77030, USA.
