# Supplementary material for: DesiRNA: structure-based design of RNA sequences with a replica exchange Monte Carlo approach
Source: Nucleic Acids Res. 2025 Jan 20;53(2):gkae1306. doi: 10.1093/nar/gkae1306 (PMC11744100; doi:10.1093/nar/gkae1306)
Supplement: gkae1306_Supplemental_Files [file gkae1306_supplemental_files.zip › DesiRNA_Supplementary_information.pdf]

Supplementary Material for

DesiRNA: structure-based design of RNA sequences with a Replica Exchange Monte Carlo approach

Tomasz K. Wirecki[1], Grzegorz Lach[1,2], Nagendar Goud Badepally[1], S. Naeim Moafinejad[1], Farhang Jaryani[1,†], Gaja Klaudel[1,2], Kalina Nec[1], Eugene F. Baulin[1], Janusz M. Bujnicki[1,*]

[1] Laboratory of Bioinformatics and Protein Engineering, International Institute of Molecular and Cell Biology in Warsaw, ul. Ks. Trojdena 4, 02-109 Warsaw, Poland
[2] Institute of Theoretical Physics, Faculty of Physics (FUW), University of Warsaw, ul. Hoża 69, 00-681 Warsaw, Poland

[*]To whom correspondence should be addressed. Tel: (+48-22) 597-07-50; Fax: (+48-22) 597-07-15; Email: janusz@iimcb.gov.pl Correspondence may also be addressed to Tomasz Wirecki, email: twirecki@iimcb.gov.pl

† Current address: Human Genome Sequencing Center, Baylor College of Medicine, Houston, TX 77030, USA

A

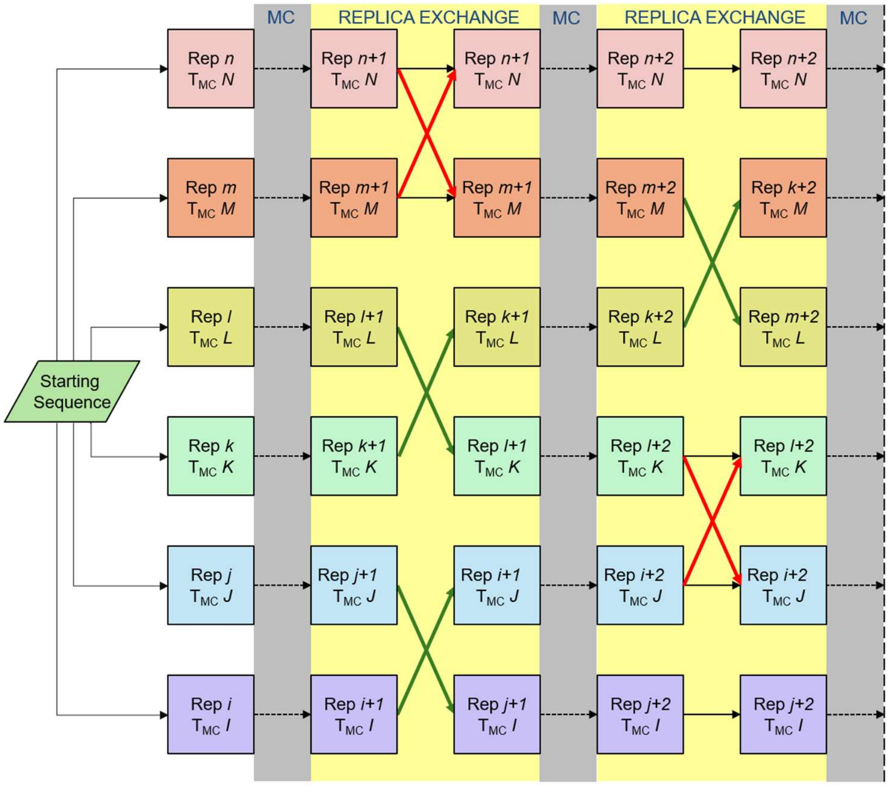

B

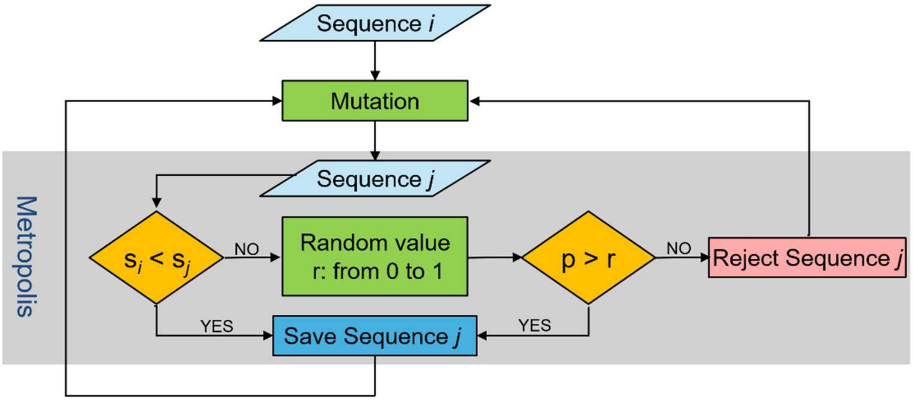

Supplementary Figure S1. Overview of the REMC algorithm as implemented in DesiRNA. (**A**) Handling of replicas. Each replica is initialized at a different T_{MC}. level and undergoes MC sampling. After every *n*th-number of MC steps, a replica exchange attempt is made, governed by equation (2) (see the main manuscript). Green arrows indicate successful exchanges, which improve sampling by allowing configurations to swap between temperature levels. Red arrows denote unsuccessful exchanges, where the configurations remain in their original replicas. (**B**) Schematic representation of the Metropolis criterion used in DesiRNA. The algorithm begins with the current Sequence *i* that undergoes a mutation step to generate new Sequence *j*. Sequence j score (*Sj*) is compared with the current Sequence *i* (*Si*). If *Sj*<*Si* (i.e., the new sequence has a lower=better score), it is automatically accepted and saved. Otherwise, the algorithm calculates an acceptance probability (p) using equation (1) (see the main manuscript).

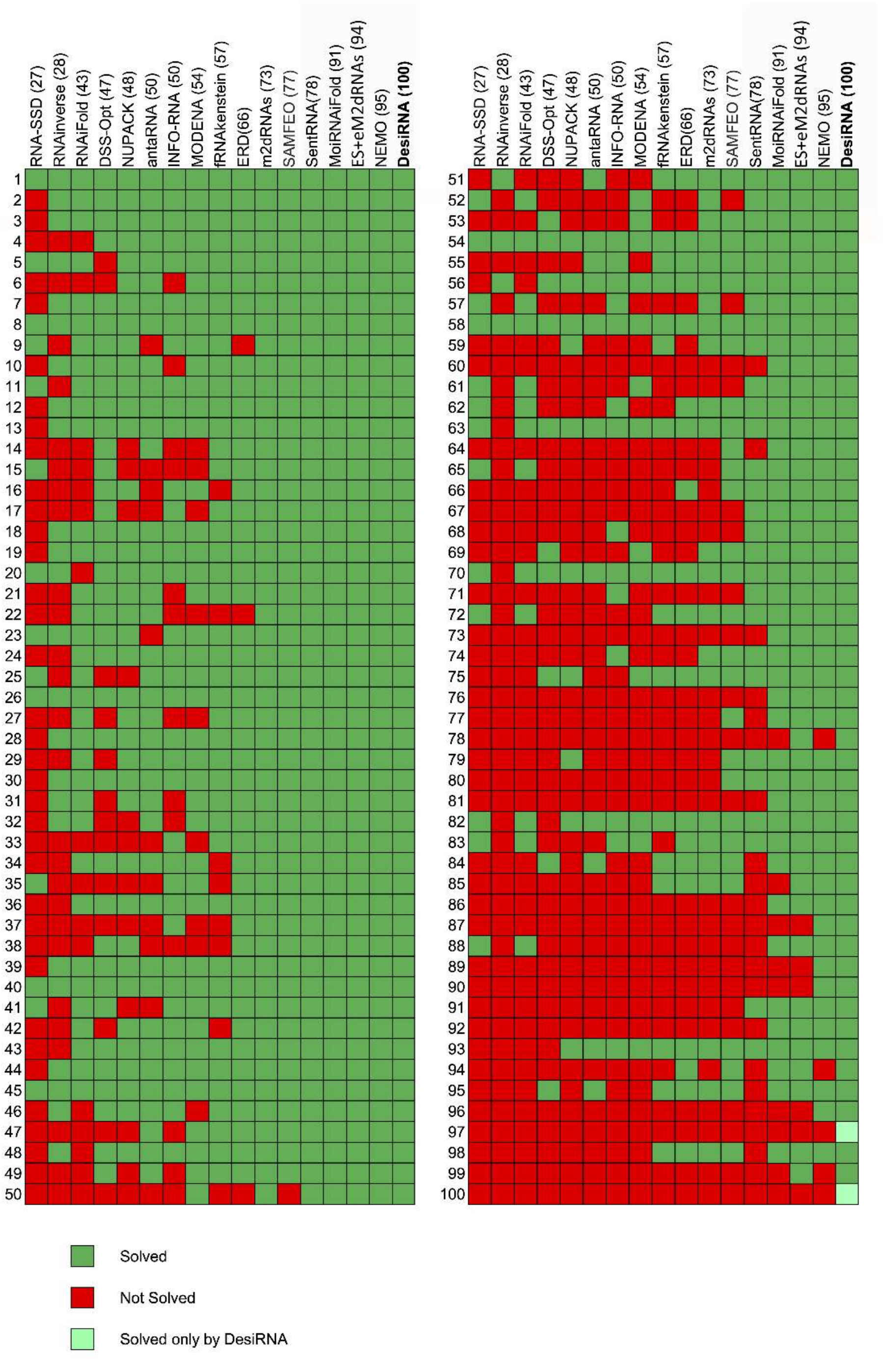

Supplementary Figure S2. Performance of the existing RNA design methods on the Eterna V1 benchmark, compared to the results of DesiRNA.

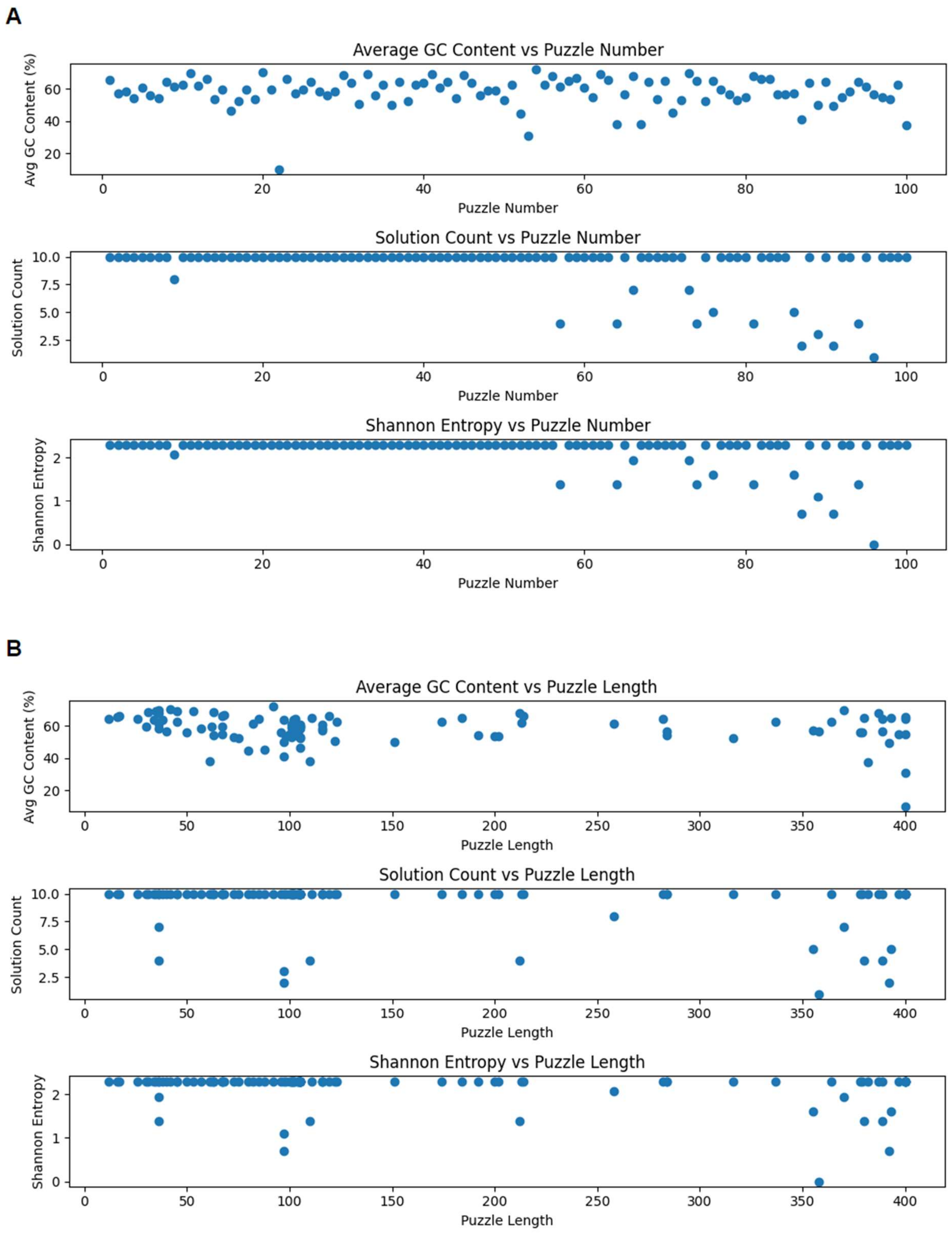

A
Average GC Content vs Puzzle Number
Avg GC Content (%)
Puzzle Number
Solution Count vs Puzzle Number
Solution Count
Puzzle Number
Shannon Entropy vs Puzzle Number
Shannon Entropy
Puzzle Number
B
Average GC Content vs Puzzle Length
Avg GC Content (%)
Puzzle Length
Solution Count vs Puzzle Length
Solution Count
Puzzle Length
Shannon Entropy vs Puzzle Length
Shannon Entropy
Puzzle Length

Supplementary Figure S3. Sequence Diversity Analysis for DesiRNA on the Eterna100 V1 Benchmark. (**A** - top) The average GC content of sequences designed for each puzzle in the Eterna100 benchmark. The GC content remains relatively consistent across puzzles, typically ranging between 40% and 60%, but shows slight variations, particularly in puzzles with higher numbers. These fluctuations reflect the different sequence design constraints imposed by the puzzle's secondary structures and base-pairing patterns. (**A** - middle) The number of distinct solutions generated for each puzzle, up to a maximum of 10. DesiRNA is able to solve most puzzles with the maximum solution count, particularly for simpler or shorter puzzles. However, for more complex puzzles (those with higher puzzle numbers), the number of solutions found tends to decrease, indicating increased difficulty or tighter design constraints. (**A** - bottom) The Shannon entropy of the solutions for each puzzle, offering a measure of sequence diversity. Higher entropy values indicate more sequence diversity. While many puzzles show consistent sequence diversity, some (especially around puzzle numbers 60-80) exhibit lower entropy values, suggesting a reduction in the diversity of possible solutions. (**B** - top) The GC content variation with puzzle length. Shorter puzzles tend to have less variation in GC content, while longer puzzles (around 300-400 nucleotides) exhibit more variability, indicating greater flexibility in sequence composition for longer structures. (**B** - middle) The number of solutions found for puzzles of varying lengths. Shorter puzzles (up to 150 nucleotides) tend to have a higher solution count, with DesiRNA frequently finding the maximum of 10 solutions. However, as puzzle length increases, the solution count begins to drop, particularly for puzzles over 300 nucleotides in length, reflecting the increased difficulty of longer structures. (**B** - bottom) The Shannon entropy (sequence diversity) across puzzle lengths. Puzzles of all lengths show a reasonable degree of diversity, but some of the longer puzzles exhibit lower entropy values, suggesting more constrained design spaces for certain larger puzzles.

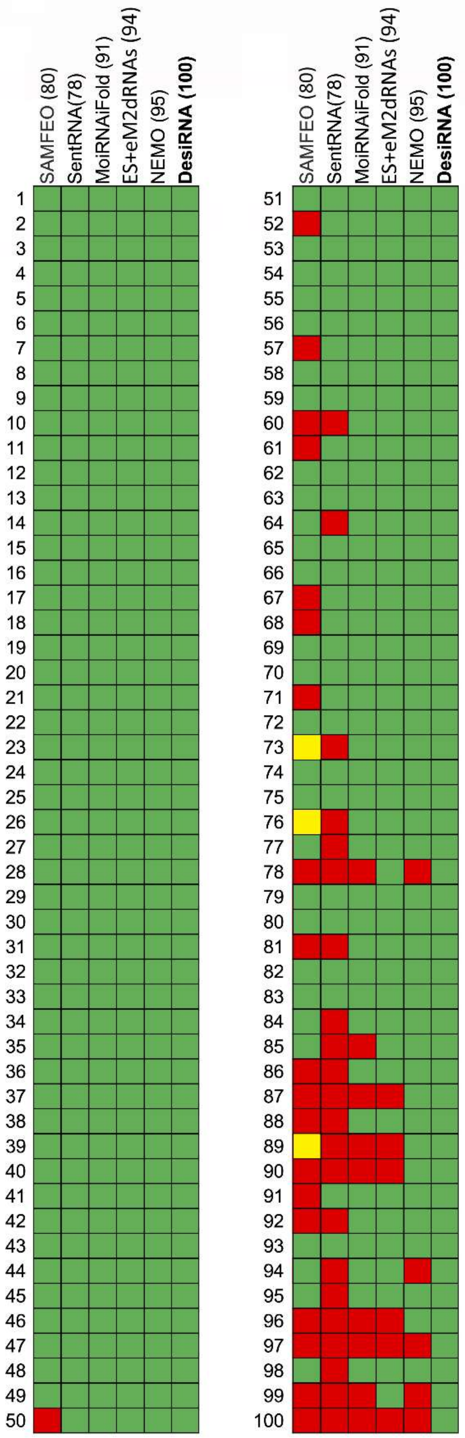

Supplementary Figure S4. Updated results for the best-performing third-party methods, where targets reported as unsolved by the authors were run on the same computational setup as DesiRNA. Only SAMFEO was able to solve additional three targets (marked in yellow), now scoring 80/100.

A

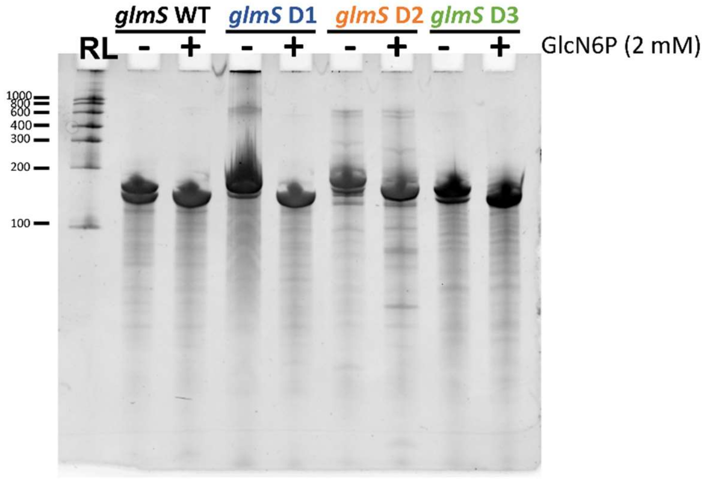

B

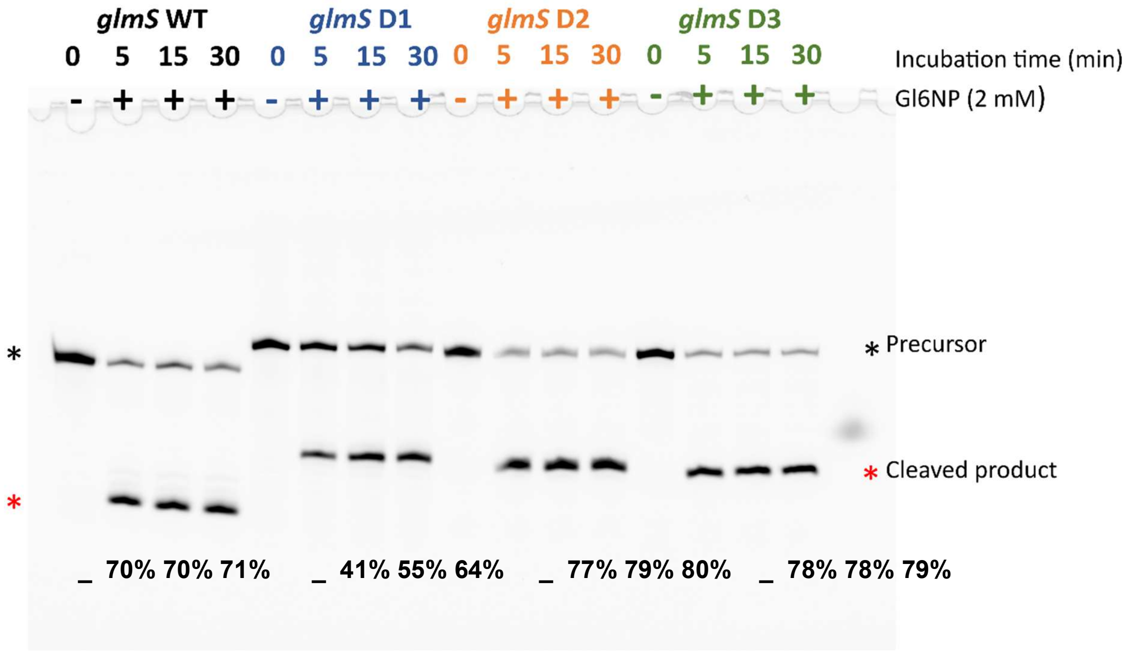

Supplementary Figure S5. Uncropped gel images of the activity assays. (**A**) Cis-cleavage activity, *in vitro* transcription (IVT) in the absence and presence of 2 mM GlN6P. The denaturing gel analysis of the IVT products shows the transcription of shorter RNA products in the presence of GlN6P. (**B**) Gel image shows the Trans-cleavage activity of DesiRNA designed *glmS* design sequences and *glmS* WT as a control.

Supplementary Table S1. Primers.

Oligo name	Oligo sequence
glmS_WT_T7_FP	AATTTAATACGACTCACTATAGGCCTTCGGGCCAAAGC
glmS_WT_EZ_T7_FP	AATTTAATACGACTCACTATAGGCCGGCTTTAAGTTGACGAG
glmS_WT_RP	TTCCTGCCCGGACTGCTCTATG
D1_T7_FP	AATTTAATACGACTCACTATAGGTGTGGAAACACAGAAG
D1_EZ_T7_FP	AATTTAATACGACTCACTATAGGAGCCTTTAGTTGACGAGGG
D1RP	TGGTGCCTCGTTGTTCTT
D2_T7_FP	AATTTAATACGACTCACTATAGGCTGGAAACAGTGAAGCGCCAGAG
D2_EZ_T7_FP	AATTTAATACGACTCACTATAGGCAGTTGAGCTGACGAGGGAAG
D2_RP	CTGCGTCTTGTTTATCTTTATCGAGCAGTC
D3_T7_FP	AATTTAATACGACTCACTATAGGCTAGTAATAGCTAAGCGCCAGGAC
D3_EZ_T7_FP	AATTTAATACGACTCACTATAGGTGCATTTAGTTGACGAGGGAAGGACTTATC
D3_EZ_T7_FP	TACCCCCGTCTTAAGCTTTATGCC

Supplementary Table S2. Cy5-labeled RNA substrates.

Substrate	Sequence	Sequence length
glmS_WT_substrate	[Cy5]GGCCUUCGGGCCAAAGCGCCUGGACUUAAAGCC	33 nt
D1_substrate	[Cy5]GGUGUGGAAACACAGAAGCGCCAGGACUAAAGGCU	35 nt
D2_substrate	[Cy5]GGCUGGAAACAGUGAAGCGCCAGAGCUCAAUUGC	34 nt
D3_substarte	[Cy5]GGCUAGUAAUAGCUAAGCGCCAGGACUAAGUGCG	34 nt

*The highlighted region corresponds to the cleaved product

Supplementary Table S3. DesiRNA solutions for the Eterna V1 benchmark (Turner1999 energy model). Attached as a separate file.

Supplementary Table S4. DesiRNA solutions for the Eterna V2 benchmark (Turner2004 energy model). Attached as a separate file.

Supplementary Table S5. RNA sequences used in the experimental analysis.

Construct name	*RNA sequence*	*Source*
glmS_WT (*T. tengcongensis* glmS ribozyme)	GGCCUUCGGGCCAAAGCGCCUGGACUUAAAGCCAUUGCACUCCGGCUUUAAGUUGACGAGGGCAGGGUUUAUCGAGACAUCGGCGGGUGCCCUGCGGUCUUCCUGCGACCGUUAGaGGACUGGUAAAACCACAGGCGACUGUGGCAUAGAGCAGUCCGGGCAGGAA	*PDB: 2Z75*
Design_1 (D1)	GGUGUGGAAACACAGAAGCGCCAGGACUAAAGGCUAAACCAAAGCAGCCUUUAGUUGACGAGGGGAGGAUUUAUCGAAGAAUCGGCGGUAUCCUUCGGCUAGGUGUCAGCCGUCAAAGUUGUUAUAAAGUCCAGAGCGAUCUGGGAAUAAAGAACAACGAGGCACCA	*DesiRNA*
Design_2 (D2)	GGCUGGAAACAGUGAAGCGCCAGAGCUCAAUUGCGAAAAACAGAGCAGUUGAGCUGACGAGGGAAGGGUUUAUCGAAACAUCGGCGGGUACCCUUCGGCCAUGUGUCGGCCGUUAAAGUUUGUACAAAAGAGUAGGCGACUGCUCGAUAAAGAUAAACAAGACGCAG	*DesiRNA*
Design_3 (D3)	GGCUAGUAAUAGCUAAGCGCCAGGACUAAGUGCGGAAAUGCAUUUAGUUGACGAGGGAAGGACUUAUCGAAAUUUCGGCGGGUGUCCUUCGGCUAGCCCCCAGCCGCUAGACUUAAGGUAAAAUCCAAAGCGAUUUGGGCAUAAAGCUUAAGACGGGGGUA	*DesiRNA*

Supplementary Table S6. Sequences designed with DesiRNA and their predicted secondary structures; constraints used in simulation; native sequence and secondary structure of 3NDB Signal Recognition Particle.

Name	Sequence	Secondary Structure Predicted by Vienna RNAfold	Secondary structure of the representative 3D models
3NDB	GUCUCGUCCCGUGGGGCUCGGCGGUGGGGGAGCA<br>UCUCCUGUAGGGGAGAUGUAACCCCCUUUACCUG<br>CCGAACCCCGCCAGGCCCGGAAGGGAGCAACGGU<br>AGGCAGGACGUCGGCGCUCACGGGGGUGCGGGAC	(((((((((((((((((.(((((..(((((.(((((<br>((((....)))))))))..)))))....((((((..<br>...(((.....(((....))).....)))..)))))<br>).))))))).))))))))...)))))))	N/A
Negative design constraints	NNNNNGNCCNNNNNNNNNNNNNGNNNNNNNNNNN<br>NNNNNNNNAGGNNNNNNNNNNNNNNNNNNNNNNN<br>NNNNANNNNNNNNAGGCNCGGAAGNGAGCANNNNN<br>NNNNNNNNCNNNNNNNNNNNNNGGNNNNCNNNNN	(((((((((((((((((.(((((..(((((.(((((<br>((((....)))))))))..)))))....((((((..<br>...(((.....(((....))).....)))..)))))<br>).))))))).))))))))...)))))))	N/A
Negative design solution	GGAGAGCCCCGCGCGCGACGAUGGAGACCCAGUA<br>GGUGCCGAAGGGCACCUAUGAGGGUCAAGAGGCC<br>AUGCACGCGGAGAGGCACGGAAGUGAGCAGCCGA<br>AGUGGCCGCAUCGCGACGCGCGGGAGAGCUCUCC	(((((((((((((((((.(((((..(((((.(((((<br>((((....)))))))))..)))))....((((((..<br>...(((.....(((....))).....)))..)))))<br>).))))))).))))))))...)))))))	N/A
Alternative structure design constraints	NNNNNNNNNNNNNNNNNNNNNNNNNNNNNNNN	((((..((((((....))))))......))))<br>((((....))))....((((((....))))))	N/A
Alternative design structure solution	GGCCCUCUGGCCGACAGGCCAGUUCCCUGGCC	((((..((((((....))))))......))))<br>((((....))))....((((((....))))))	((((..((((((....))))))..<br>....))))<br>((((....))))....((((((..<br>..))))))
Monomer design constraints	GADNNHCNNNGADNNHCNN&GADNNHCNNNGADN<br>NHCNN	..((((......))))...&..((((......))))<br>...	N/A
Dimer design constraints	GADNNHCNNNGADNNHCNN&GADNNHCNNNGADN<br>NHCNN	((((((((.((((((((&)))))))).))))))<br>)))	N/A
Monomer design solution	GAGCGUCAAAGAGCGCCCC&GAGCGUCAAAGAGC<br>GCCCC	..((((......((((...&..))))......))))<br>...	(.((((......)))).).&(.((<br>((......)))).).
Dimer design solution	GAGGAUCUCGGAGAUCCUC&GAGGAUCUCGGAGA<br>UCCUC	((((((((.((((((((&)))))))).))))))<br>)))	((((((((.((((((((&))))<br>)))).)))))))))
